# Decreased under 5 mortality and improved in-hospital clinical care after ETAT+ training and implementation at a pediatric emergency unit in Eastern Uganda: a quasi- experimental study

**DOI:** 10.21203/rs.3.rs-10893480/v1

**Published:** 2026-10-22

**Authors:** Joan Wamulugwa, Faith Oguttu, Ritah Nantale, Joel Okwir, Julian Abeso, Martin Chebet, Alain Nyalihama, John Obbo, Esther Nabutonyela, Mark Nangoli, Agnes Napyo, David Mukunya, Milton W Musaba, Peter Olupot Olupot, Sarah Kiguli

## Abstract

Background At 52 deaths per 1000 live births, the burden of under 5 mortality in Uganda is more than twice the sustainable development goal target of 25 deaths per 1000 live births by 2030. The high under 5 mortality in Uganda could be attributed to unsupervised implementation of life-saving guidelines. We aimed to evaluate the effect of Emergency Triage Assessment and Treatment Plus admission care (ETAT+) guidelines refresher training and its implementation on clinical care, mortality, and length of hospital stay among children under five years. Methods We conducted a quasi-experimental study from September to October 2024 as the pre-implementation phase. Health care workers underwent a three-day refresher training in the ETAT+ guidelines. During the implementation phase we ensured availability of supplies to do emergency assessment. We collected data from November to December 2024 during the post‑implementation phase. We compared in-hospital clinical care, mortality and duration of in-hospital stay among children under 5 years between the pre and post-implementation periods. We conducted generalized linear regression models of the binomial family with a log link and robust variance estimation to estimate prevalence ratios. Results We enrolled 1402 children, 696 during the pre-implementation phase and 707 during the post implementation phase. The intervention was associated with an increase in the likelihood of children having emergency vitals and investigations done and documented after the training: temperature [Adjusted Prevalence Ratio (APR) 3.93, 95% Confidence Interval (CI) (3.45–4.49) p < 0.001], oxygen saturation [APR3.56, 95% CI (3.04,4.15) p < 0.001], malaria-test [APR1.33, 95% CI (1.26,1.41) p < 0.001], hemoglobin estimation [APR 1.81, 95% CI (1.56,2.12) p < 0.001]. The intervention was also associated with children being less likely to have a prolonged hospital stay [APR 0.88, 95% CI (0.85,0.91) P < 0.001]. The intervention was not associated with significant differences in mortality [APR 0.71, 95% CI (0.37,1.36) p = 0.300]. Conclusion ETAT+ training and implementation was associated with improved clinical care and outcomes among children under five years. We recommend regular refresher ETAT+ trainings and implementation of ETAT+ guidelines at pediatrics emergency units. Given our study design, our findings are only suggestive of association and not causation, and need to be interpreted with caution. Clinical trial number : not applicable.

